# Prevalence and Serotype Diversity of *Salmonella enterica* in the Estonian Meat Production Chain in 2016–2020

**DOI:** 10.3390/pathogens10121622

**Published:** 2021-12-14

**Authors:** Kaisa Kuus, Toomas Kramarenko, Jelena Sõgel, Mihkel Mäesaar, Maria Fredriksson-Ahomaa, Mati Roasto

**Affiliations:** 1 Chair of Food Hygiene and Veterinary Public Health, Institute of Veterinary Medicine and Animal Sciences, Estonian University of Life Sciences, Fr. R. Kreutzwaldi 56-3, 51006 Tartu, Estonia; kaisa.kuus@student.emu.ee (K.K.); mihkel.maesaar@emu.ee (M.M.); 2 Veterinary and Food Laboratory, Fr. R. Kreutzwaldi 30, 51006 Tartu, Estonia; toomas.kramarenko@vetlab.ee; 3 Agriculture and Food Board, Väike-Paala 3, 11415 Tallinn, Estonia; jelena.sogel@pta.agri.ee; 4 Department of Food Hygiene and Environmental Health, Faculty of Veterinary Medicine, University of Helsinki, Agnes Sjöbergin katu 2, P.O. Box 66, 00014 Helsinki, Finland; maria.fredriksson-ahomaa@helsinki.fi

**Keywords:** surveillance program, *Salmonella enterica*, prevalence, serotypes, meat production chain, human salmonellosis

## Abstract

Background: *Salmonella enterica* represents a considerable public concern worldwide, with farm animals often recognised as an important reservoir. This study gives an overview of the prevalence and serotype diversity of *Salmonella* over a 5-year period in the meat production chain in Estonia. Data on human salmonellosis over the same period are provided. Methods: *Salmonella* surveillance data from 2016 to 2020 were analysed. Results: The prevalence of *Salmonella* at the farm level was 27.7%, 3.3% and 0.1% for fattening pigs, cattle and poultry, respectively. *S*. Derby was the most prevalent serotype at the farm level for fattening pigs and *S*. Dublin for cattle. The top three serotypes isolated at the slaughterhouse and meat cutting levels were *S*. Derby, monophasic *S*. Typhimurium and *S*. Typhimurium with proportions of 64.7%, 9.4% and 7.0%, respectively. These serotypes were the top five most common *Salmonella* serotypes responsible for human infections in Estonia. *S*. Enteritidis is the main cause (46.9%) of human salmonellosis cases in Estonia, but in recent years, Enteritidis has not been detected at the slaughterhouse or meat cutting level. Conclusion: In recent years, monophasic *S*. Typhimurium has become epidemiologically more important in Estonia, with the second-highest cause in human cases and third-highest among the most prevalent serotypes of *Salmonella enterica* in the meat chain.

## 1. Introduction

Salmonellosis is the second-most commonly reported zoonosis in the European Union (EU) and represents a major public health concern [1,2]. According to the European Food Safety Authority (EFSA) and European Centre for Disease Prevention and Control (ECDC) annual reports of zoonotic diseases 2018 and 2019, the trend for salmonellosis in humans in the EU has stabilised over recent years after a long period of a declining trend [1,2]. The notification rate of salmonellosis was 20.0 confirmed cases per 100,000 population, with 90,105 total cases of salmonellosis in humans in the EU in 2019 [2]. In 2018, the notification rate was almost at the same level, with 20.1 cases per 100,000 population [1]. The three most commonly reported serotypes isolated from humans in 2019 were *S*. Enteritidis, *S*. Typhimurium and its monophasic variant 1,4,[5],12:i:-, which accounted for 70.4% of all confirmed human cases in the EU [2]. A total of 154 salmonellosis cases (150 confirmed cases with an incidence rate of 11.3 per 100,000 residents) were registered in Estonia in 2019 compared to 323 salmonellosis cases in 2018. Estonian salmonellosis cases were mostly attributed to the serotypes Enteritidis (40.1%) and monophasic *Salmonella* Typhimurium (16.2%). Moreover, *Salmonella* was the main cause of foodborne outbreaks in Estonia, with nine reported outbreaks in 2019 [3]. In 2020, fewer cases of salmonellosis were registered, which was probably due to the impact of the COVID-19 pandemic [4].

Human salmonellosis is commonly caused by the inappropriate handling and/or consumption of contaminated food. *Salmonella* spp. in humans may cause gastroenteritis and, in rare cases, complications such as bacteraemia and reactive arthritis [5]. *Salmonella* has often been detected in meat and meat products. In only a few countries, a very low *Salmonella* prevalence in the meat production chain has been reported; for example, in Finland, no *Salmonella* were found in carcass swabs or pork during the 2010s [5]. Generally, in the EU, the overall prevalence of *Salmonella* in non-ready-to-eat (RTE) and RTE meat and meat products was reported to be 1.7% and 0.6%, respectively [2]. According to the EFSA and ECDC (2021), most of the *Salmonella*-positive samples from the entire meat production chain in the EU were found to be from fresh poultry and fresh pig meats. The rising prevalence of monophasic *S*. Typhimurium has been reported in many studies. This serotype has often been associated with pigs, fresh pork and semi-finished meat products intended to be eaten cooked [6,7,8].

Since the reported incidence rates of human *Salmonella* infections are continuously high, and meat and meat products are widely consumed food items in Estonia, the aim of this study was to determine the *Salmonella* prevalence and related serotypes in the meat production chain under *Salmonella* control and surveillance programmes in Estonia during the period 2016–2020. Additionally, data on human *Salmonella* infections in the same period are presented.

## 2. Results

### 2.1. Salmonella at Farm Level

The *Salmonella* prevalence at the fattening pig, cattle and broiler chicken farm levels is presented in Table 1. In 2016–2020, 119 fattening pig farms were sampled, and 27.7% (*n* = 33) were *Salmonella*-positive. Among the *Salmonella* isolates, the most prevalent serotype was *S*. Derby (*n* = 25; 75.8%), followed by *S*. Typhimurium and its monophasic variant 15.2% (*n* = 5). Additionally, *S*. Agona (2), *S*. Cholerasuis (2), *S*. Mbandaka (1) and *S*. Dublin (1) were isolated at Estonian fattening pig farms.

In the framework of the *Salmonella* control programme of Estonia, a total of 583 cattle farms were sampled for *Salmonella*, of which 19 (3.3%) were positive in 2016–2020 (Table 1). *S*. Typhimurium, including the monophasic variant, was the most often isolated serotype (50.0%), followed by *S*. Dublin (40.0%). 

During 2016–2020, a total of 3187 broiler chicken flocks were sampled for *Salmonella*, and three (0.1%) of the flocks were positive. Serotyping of these isolates detected *S*. Typhimurium, *S*. Infantis and *S*. Derby.

### 2.2. Salmonella spp. at Slaughterhouse Level

At the Estonian slaughterhouse level within the five-year period, a total of 3060 samples were taken, and the overall prevalence of *Salmonella* was 2.2% (Table 2). The proportion of *Salmonella*-positive samples was the highest for pigs, of which 3.2% (*n* = 61) of the sampled carcases were found to be positive for *Salmonella*. The most prevalent serotype (78.7%) among the *Salmonella* isolates obtained from pig carcasses was *S*. Derby (Table 3). The *Salmonella* prevalence in cattle carcasses was low, with two (0.2%) *Salmonella*-positive samples within the five-year period. The cattle-related serotypes were determined to be *S*. Dublin and *S*. Altona (Table 3). All broiler chicken carcass samples were negative for *Salmonella*, but monophasic *S*. Typhimurium and *S*. Typhimurium were found from four quail carcass samples. However, quail meat production in Estonia is very small, and all samples originated from one enterprise where the slaughtering, meat cutting and processing of quails were performed onsite.

### 2.3. Salmonella spp. at Meat Cutting Level

The *Salmonella* prevalence at the meat cutting level is shown in Table 4. Similarly, at the slaughterhouse level, the most *Salmonella* isolates were obtained from pigs. Altogether, 14 samples (1.1%) from 1290 fresh pork samples were *Salmonella*-positive, and *S*. Derby (50.0%) was the most prevalent serotype during 2016–2020. In the same period, only two samples (0.4%) from 556 fresh beef samples were found to be positive for *Salmonella* at the meat cutting level (Table 4). The cattle-associated serotypes were *S*. Dublin and *S*. Mbandaka (Table 3). All fresh broiler chicken meat samples were negative for *Salmonella*. Monophasic *S*. Typhimurium was isolated from two positive quail meat samples.

### 2.4. Human Salmonella Infections

A total of 1204 human salmonellosis cases were reported by the Estonian Health Board in 2016–2020 (Table 5). The salmonellosis notification rate in the pre-COVID period during 2015–2019 in Estonia was 18.8 on average, which is comparable to the EU notification rate of 20.3 reported for the same period. During 2015–2019, the highest number of disease cases was reported in the year 2016, when 358 confirmed salmonellosis cases were reported in Estonia with a notification rate of 27.3 per 100,000 population, and the lowest in the year 2020, when 92 salmonellosis cases were reported with a notification rate of 6.9 per 100,000 population. Thus, the number of *Salmonella* human infections in Estonia varied greatly from year to year and was influenced by the number of *Salmonella* outbreaks. The most prevalent *Salmonella* serotype in the human *Salmonella* infections was *S*. Enteritidis (46.9%), followed by *S*. Typhimurium (16.8%), *S*. Infantis (10.5%), monophasic *S*. Typhimurium (7.1%) and *S*. Derby, with 1.2% of all salmonellosis cases in the investigated study period in Estonia (Table 5) [4,9].

## 3. Discussion

*Salmonella enterica* is a major food-borne pathogen worldwide, and salmonellosis is one of the most commonly reported gastrointestinal infection in humans and an important cause of foodborne outbreaks in the EU [2]. As with the EU, salmonellosis is the second-most commonly reported gastrointestinal infection in humans in Estonia and the main cause of foodborne outbreaks [4]. The most prevalent serotypes causing human salmonellosis cases in Estonia are *S*. Enteritidis, *S*. Typhimurium and its monophasic variant. *S*. infantis, which was associated with a large outbreak of 88 cases in 2016 in Estonia and ranked third in human salmonellosis cases during the period 2016–2020. However, in 2017–2020, few *Salmonella* infections were caused by *S*. Infantis per year (Table 5). Most *Salmonella* human infections in Estonia are seasonal, with the peak incidence occurring from April to October, and the trend is remarkably influenced by the number and size of the outbreaks. During 2016–2020, 54 outbreaks of salmonellosis were reported, with 414 disease cases in Estonia. Most of the outbreaks were caused by *S*. Enteritidis, *S*. Typhimurium and monophasic *S*. Typhimurium. The incidences per outbreak caused by *S*. Enteritidis were greater than for the other serotypes [4].

The Estonian *Salmonella* control programme involves the farm, slaughterhouse and the meat cutting level. It aims to prevent and eradicate salmonellosis in production animals and to protect humans from zoonotic diseases transmitted through animals, feed and food.

In the present study, *S*. Derby was the most often isolated serotype. It was found in samples originating from all stages of pork production but mostly at pig farms and at the slaughterhouse level. The incidence of human infection caused by this serotype appears to be modest over the period, with few salmonellosis cases per year in Estonia. However, the inappropriate handling and cooking of raw semi-final meat products, especially pork products, can be a source of *Salmonella* infections. It is known that, despite the high prevalence of *Salmonella* in pig carcasses and in raw pork, *S*. Derby does not cause significant enteric disease in pigs [10]; nevertheless, pork contaminated with *S*. Derby may cause human *Salmonella* infections. In Germany, *S*. Derby has been ranked as the fourth- to fifth-most common cause of *Salmonella* outbreaks in humans [11]. *S*. Derby has also been found to have a high epidemiological importance in China, which is the largest pork consumer country in the world [12]. *S.* Derby is one of the top five most common *Salmonella* serotypes responsible for human infections during the five-year period of 2016–2020 in Estonia (Table 5). Additionally, according to an Estonian zoonoses report [13], *S*. Derby was the most prevalent *Salmonella* serotype in food samples at the retail level in 2011–2014 and 2016–2018. Importantly, in recent years, the majority of *Salmonella*-positive food samples in Estonia have been obtained from raw pork and pork products [13]. Similarly, *S*. Derby has been found to be the dominant serotype in pork in many other countries [7,14,15].

In Estonia, monophasic *S*. Typhimurium is very frequent among serotypes of human origin after infections caused by *S*. Enteritidis and biphasic *S*. Typhimurium. Animals, often pigs, are considered the main source of a monophasic variant of *S*. Typhimurium, and most commonly, the infections are associated with the consumption of fresh pork or beef [13,15,16,17]. The percentage of monophasic *S*. Typhimurium in human salmonellosis increased from 3.6% in 2016 to 10.9% in 2020 (Table 5). The growing number of monophasic *S*. Typhimurium strains isolated from human patients indicates the recent emergence of this serotype in the Estonian food chain. In the present study, *Salmonella* 1,4,[5],12:i:- was the second most common *Salmonella* serotype isolated from the slaughterhouse and meat cutting levels in Estonia (Table 3). In Estonia’s neighbouring country Latvia, the most prevalent (36%) serotypes in meat and meat products were also *S*. Typhimurium and the monophasic variant of *S*. Typhimurium [18]. The monophasic variant of S. Typhimurium has become one of the most common *Salmonella* serotypes in the pig food chain in Europe and the United States, with a link between human infections and the consumption of pork and pork products [19,20]. The contamination of pork with monophasic *S*. Typhimurium has been reported in many studies [21,22,23,24]. The emergence of this serotype has not only been reported in the EU and the USA but all over the world in the last decade. Accordingly, Sun et al. [20] found that monophasic *S*. Typhimurium has successfully spread worldwide, with high infection rates and broad antibiotic resistance, with the pig considered an important reservoir. The worldwide distribution of monophasic *S*. Typhimurium can be explained by the fact that, in addition to the pork chain, this serotype has been isolated from many other sources, including cattle, companion animals, humans and the environment [20,25]. The emergence of monophasic *S*. Typhimurium in Estonia could also be related to the consumption preference of pork meat and pork products among consumers. Pork is the most consumed meat in Estonia. In 2020, 40 kg of pork was consumed per inhabitant, followed by 27.2 kg of poultry meat [26]. The proportion of pork-related monophasic *S*. Typhimurium among the isolates obtained from the slaughterhouse and meat cutting levels of Estonia is low (Table 4), but almost 20% of pork consumed is imported into Estonia, mainly from Germany and Poland [26], and monophasic *S*. Typhimurium is predominant in pigs in Poland [27]. A recent study [28] found that the most prevalent serotype in pork was *S.* typhimurium in Romania, while a study in Czechia found that *S*. typhimurium and its monophasic variant were the predominant serotypes there in pork meat [29]. The epidemiologic success of monophasic *S*. Typhimurium has been explained by the emergence and expansion of new epidemic clones. In the United Kingdom, the monophasic epidemic clones showed a novel genomic island encoding a resistance to heavy metals and a composite transposon-encoding antimicrobial drug resistance gene, which was linked to their epidemiologic success during an epidemic [30]. A very low prevalence of *Salmonella* in the whole broiler chicken meat production chain may be related to the fact that there is only one large-scale broiler chicken company in Estonia. This company implements a vertically integrated meat safety assurance system that covers the entire meat production chain, including feed production, farms, slaughterhouse, meat cutting and a processing plant. This vertically integrated system enables the in-house sharing of data in real-time, allowing prompt risk mitigation measures to be taken at any production step where they are applicable. 

Whole-genome and phylogenomic analyses for *Salmonella* isolates were not carried out in this study, because during the entire 2016–2020 period, the systematic application of whole-genome sequencing (WGS) in routine *Salmonella* monitoring was not yet established in Estonia. However, recently, laboratory competence for WGS analyses was established at the Veterinary and Food Laboratory of Estonia.

## 4. Materials and Methods

### 4.1. Sample Collection

Samples at different stages of the meat production chain were collected during the five-year period of 2016–2020 in Estonia. In this cross-sectional study, the samples were taken within the framework of the national *Salmonella* control and surveillance programme of the competent authority and analysed at the state veterinary and food laboratory. Broiler chicken farms were sampled by a competent authority and by food business operators.

In total, 119 fattening pig, 583 cattle herds and 3187 poultry flocks were sampled during 2016–2020 to determine the *Salmonella* prevalence and related serotypes at the farm level. In total, 3060 and 1926 samples were taken at the slaughterhouses and meat cutting plants, respectively. In accordance with the Estonian *Salmonella* control programme at the farm level, approximately 1/5 of the pig and cattle herds were examined based on a risk-based approach. Faecal samples were taken on the farms. At the slaughterhouses, pig and cattle carcass surface samples were taken using the abrasive sponge method. Neck skins of poultry carcases were sampled. At the meat cutting level, fresh meat from the meat cutting plant or cuts of meat resulting from its processing were taken from a processing line or from another suitable place. The sampling rules described in Regulation (EC) No 2073/2005 on the microbiological criteria for foodstuffs were followed. The analyses were performed at the Veterinary and Food Laboratory of Estonia. Additionally, Estonian Health Board data on human salmonellosis cases during 2016–2020 and related serotypes were presented.

### 4.2. Isolation and Identification of Salmonella

*Salmonella* was isolated and identified as described in the standard method ISO 6579. In brief, a pre-enrichment step in Buffered Peptone Water (BPW) at 37 ± 1 °C for 18 ± 2 h was used, followed by selective enrichment in Müller-Kauffmann Tetrathionate Novobiocin Broth (MKTTn) at 37 ± 1 °C and Rappaport-Vassiliadis Soya peptone broth (RVS) 41.5 ± 0.5 °C. Modified Semi-solid Rappaport-Vassiliadis (MSRV) agar was used instead of RVS when faecal samples from farms were analysed. For isolation, selective Xylose Lysine Deoxycholate (XLD) agar and Brilliant Green (BG) agar plates were used, which were incubated at 37 ± 1 °C for 24 ± 3 h. The characteristic colonies were subcultured and confirmed by biochemical and serological tests according to ISO 6579. All microbiological media mentioned above originated from Biolife Italiana s.r.l.—Mascia Brunelli S.p.A., Milano, Italy.

### 4.3. Serological Confirmation

Serotyping *Salmonella* isolates originating from the meat chain was performed at the Veterinary and Food Laboratory. Identification to the serovar level was performed by the Kauffmann-White-Le Minor scheme using commercially available antisera (Statens Serum Institut, Copenhagen, Denmark). The lacking phase 2 flagellar antigen of the monophasic variant of *Salmonella* Typhimurium was initially verified using the flagellar-phase reversal method following definitive confirmation by the PCR method, as described by the EFSA Panel of Biological Hazards [31]. All salmonellosis cases diagnosed by diagnostic or hospital laboratories are notifiable and must be reported to Health Board of Estonia. However, the *Salmonella* spp. isolates were sent to the Central Laboratory of Health Board on a voluntary basis, which performed a serological confirmation routinely.

### 4.4. Statistical Analyses

Confidence intervals (CI) of the proportions with Yates’ continuity correction were calculated using the prop.test function included in Statistical Package R v3.6.3 (R Foundation for Statistical Computing, Vienna, Austria). 

## 5. Conclusions

*Salmonella enterica* is one of the most important zoonotic agents causing foodborne enteric diseases in Estonia. This survey provides useful insight into the *Salmonella* prevalence and circulating serotypes in the meat production chain over a 5-year period in Estonia. *S.* Derby was the most prevalent *Salmonella* serotype in the Estonian meat production chain isolated from fattening pigs at the farm level, from pig carcasses at slaughterhouses and fresh pork at meat cutting plants. The Estonian *Salmonella* surveillance programme covers retail-level sampling, and *S*. Derby was the most frequently detected *Salmonella* serotype from raw pork and pork products. *S*. Derby was the fifth-most common cause of human *Salmonella* infections in Estonia. However, most *Salmonella* human infections in Estonia are caused by *S*. Enteritidis and *S*. Typhimurium. In recent years, the monophasic variant of *S*. Typhimurium has emerged regarding *Salmonella* human infections in Estonia. This meat chain study indicates the epidemiological importance of monophasic *S*. Typhimurium, *S*. Typhimurium and *S*. Derby in Estonia.

## Figures and Tables

**Table 1:** *Salmonella* prevalence at the farm level during 2016–2020 in Estonia.

Year	Pig			Cattle			Broiler Chicken		
	Studied Herds ^a^	Positive Herds		Studied Herds ^b^	Positive Herds		Studied Flocks ^c^	Positive Flocks	
	(*n*)	(*n*)	(%)	(*n*)	(*n*)	(%)	(*n*)	(*n*)	(%)
2016	17	1	5.9	144	2	1.4	732	0	0.0
2017	25	7	28.0	143	5	3.5	600	1	0.2
2018	22	6	27.3	89	3	3.4	596	0	0.0
2019	29	13	44.8	107	3	2.8	600	2	0.3
2020	26	6	23.1	100	6	6.0	659	0	0.0
Total	119	33	27.7 95% CI 20.1–36.8	583	19	3.3 95% CI 2.0–5.1	3187	3	0.09 95% CI 0.02–0.3

^a^ Herd level, fattening pigs; samples taken by the Veterinary and Food Board in the framework of the *Salmonella* monitoring programme of Estonia. ^b^ Samples taken by the Veterinary and Food Board in the framework of the *Salmonella* control programme of Estonia. ^c^ Samples taken by the Veterinary and Food Board and by the Food Business Operator.

**Table 2:** *Salmonella* prevalence at the slaughterhouse level during 2016–2020 in Estonia.

Carcass Type	2016			2017			2018			2019			2020			Total			
	Total	Positive		Total	Positive		Total	Positive		Total	Positive		Total	Positive		Total	Positive		
	(*n*)	(*n*)	(%)	(*n*)	(*n*)	(%)	(*n*)	(*n*)	(%)	(*n*)	(*n*)	(%)	(*n*)	(*n*)	(%)	(*n*)	(*n*)	(%)	(95% CI)
Pig	335	12	3.6	403	7	1.7	398	14	3.5	401	15	3.7	370	13	3.5	1907	61	3.2	2.5–4.1
Cattle	211	0	0.0	209	1	0.5	215	0	0.0	214	1	0.5	212	0	0.0	1061	2	0.2	0.03–0.8
Broiler chicken	16	0	0.0	16	0	0.0	14	0	0.0	12	0	0.0	12	0	0.0	70	0	0.0	0.0–6.5
Quail	- ^a^	- ^a^	- ^a^	- ^a^	- ^a^	- ^a^	6	2	33.3	8	2	25.0	8	0	0.0	22	4	18.2	6.0–41.0
Total	562	12	2.1	628	8	1.3	633	16	2.5	635	18	2.8	602	13	2.2	3060	67	2.2	1.7–2.8

^a^ No samples.

**Table 3:** Distribution of *Salmonella* spp. serotypes obtained at the slaughterhouse and meat cutting levels during 2016–2020 in Estonia.

*Salmonella* Serotype	Slaughterhouse			Meat Cutting			Total		
	Pig	Cattle	Poultry	Pig	Cattle	Poultry			
	(*n*)	(*n*)	(*n*)	(*n*)	(*n*)	(*n*)	(*n*)	(%)	(95% CI)
Derby	48	0	0	7	0	0	55	64.7	53.5–74.6
Typhimurium 1,4[5],12:i:-	2	0	3 ^a^	1	0	2 ^b^	8	9.4	4.4–18.2
Typhimurium	3	0	1 ^a^	2	0	0	6	7.0	2.9–15.3
Infantis	2	0	0	1	0	0	3	3.5	0.9–10.7
Agona	3	0	0	0	0	0	3	3.5	0.9–10.7
Mbandaka	1	0	0	0	1	0	2	2.4	0.4–9.0
Dublin	0	1	0	0	1	0	2	2.4	0.4–9.0
Bredeney	2	0	0	0	0	0	2	2.4	0.4–9.0
Altona	0	1	0	0	0	0	1	1.2	0.06–7.3
*Salmonella enterica* subsp. *enterica* (- ; f, g ; -)	0	0	0	3	0	0	3	3.5	0.9–10.7
Total (%)	71.7 (*n* = 61)	2.4 (*n* = 2)	4.7 (*n* = 4)	16.4 (*n* = 14)	2.4 (*n* = 2)	2.4 (*n* = 2)	100.0 (*n* = 85)	100.0	

^a^ Quail. ^b^ Quail meat.

**Table 4:** *Salmonella* prevalence at the meat cutting level during 2016–2020 in Estonia.

Animal Species	2016			2017			2018			2019			2020			Total			
	Total	Positive		Total	Positive		Total	Positive		Total	Positive		Total	Positive		Total	Positive		
	(*n*)	(*n*)	(%)	(*n*)	(*n*)	(%)	(*n*)	(*n*)	(%)	(*n*)	(*n*)	(%)	(*n*)	(*n*)	(%)	(*n*)	(*n*)	(%)	(95% CI)
Pig	250	4	1.6	252	1	0.4	272	3	1.1	276	4	1.5	240	2	0.8	1290	14	1.1	0.6–1.9
Cattle	106	0	0.0	102	0	0.0	112	1	0.9	120	1	0.8	116	0	0.0	556	2	0.4	0.1–1.4
Broiler chicken	12	0	0.0	12	0	0.0	16	0	0.0	12	0	0.0	12	0	0.0	64	0	0.0	0.0–1.7
Quail	- ^a^	- ^a^	- ^a^	- ^a^	- ^a^	- ^a^	- ^a^	- ^a^	- ^a^	8	2	25.0	8	0	0.0	16	2	12.5	2.2–39.6
Total	368	4	1.1	366	1	0.3	400	4	1.0	416	7	1.7	376	2	0.5	1926	18	0.9	0.6–1.5

^a^ No samples.

**Table 5:** *Salmonella* spp. serotypes in humans in Estonia between 2016 and 2020.

*Salmonella* Serotype	Number of Disease Cases					Total	
	2016	2017	2018	2019	2020		
	(*n*)	(*n*)	(*n*)	(*n*)	(*n*)	(*n*)	(%)
Enteritidis	122	124	213	63	43	565	46.9
Typhimurium	74	78	15	23	13	203	16.8
Infantis	113	6	6	1	1	127	10.5
1,4[5],12:i:-	13	25	13	25	10	86	7.1
Derby	2	3	3	4	2	14	1.2
Java	0	1	4	0	2	7	0.6
Sandiego	0	0	3	0	0	3	0.3
Virchow	0	1	3	2	0	6	0.5
Thompson	3	0	1	0	2	6	0.5
Stanley	1	0	3	0	0	4	0.3
Mbandaka	1	3	0	0	0	4	0.3
Oranienburg	1	0	1	2	0	4	0.3
Coeln	0	2	2	0	0	4	0.3
*S.* C group	3	2	0	0	0	5	0.4
*S.* B and D groups	3	3	8	0	3	17	1.4
*Salmonella* spp.	9	17	24	14	9	73	6.1
All other rare serotypes	13	14	24	20	7	78	6.5
Total (%)	29.7 (*n* = 358)	23.1 (*n* = 279)	26.8 (*n* = 323)	12.8 (*n* = 154)	7.6 (*n* = 92)	100.0 (*n* = 1206)	100.0

## Data Availability

Not applicable.
